# Amnestic mild cognitive impairment in Parkinson’s disease: White matter structural changes and mechanisms

**DOI:** 10.1371/journal.pone.0226175

**Published:** 2019-12-12

**Authors:** Fuyong Chen, Tao Wu, Yuejia Luo, Zhihao Li, Qing Guan, Xianghong Meng, Wei Tao, Haobo Zhang

**Affiliations:** 1 Department of Neurosurgery, Shenzhen University General Hospital, Shenzhen University, Shenzhen, Guangdong Province, China; 2 Shenzhen University Clinical Research Center for Neurological Diseases, Shenzhen, Guangdong Province, China; 3 Department of Neurosurgery, the First Affiliated Hospital of Fujian Medical University, Fuzhou, Fujian Province, China; 4 Department of Neurology, National Clinical Research Center for Geriatric Disorders, Beijing Institute of Geriatrics, Xuanwu Hospital, Capital Medical University, Beijing, China; 5 Beijing Key Laboratory on Parkinson's Disease, Parkinson Disease Center of Beijing Institute for Brain Disorders, Beijing, China; 6 School of Psychology, Shenzhen University, Shenzhen, Guangdong Province, China; 7 Shenzhen Key Laboratory of Affective and Social Cognitive Science, Shenzhen, Guangdong Province, China; 8 Center for Emotion and Brain, Shenzhen Institute of Neuroscience, Shenzhen, Guangdong Province, China; Anhui Medical University, CHINA

## Abstract

Mild cognitive impairment (MCI) is a heterogeneous cognitive disorder that is often comorbid with Parkinson’s diseases (PD). The amnestic subtype of PD-MCI (PD-aMCI) has a higher risk to develop dementia. However, there is a lack of studies on the white matter (WM) structural changes of PD-aMCI. We characterized the WM structural changes of PD-aMCI (n = 17) with cognitively normal PD (PD-CN, n = 19) and normal controls (n = 20), using voxel-based and tract-based spatial statistics (TBSS) analyses on fractional anisotropy (FA) axial diffusivity (AD), and radial diffusivity (RD). By excluding and then including the motor performance as a covariate in the comparison analysis between PD-aMCI and PD-CN, we attempted to discern the influences of two neuropathological mechanisms on the WM structural changes of PD-aMCI. The correlation analyses between memory and voxel-based WM measures in all PD patients were also performed (n = 36). The results showed that PD-aMCI had smaller FA values than PD-CN in the diffuse WM areas, and PD-CN had higher AD and RD values than normal controls in the right caudate. Most FA difference between PD-aMCI and PD-CN could be weakened by the motor adjustment. The FA differences between PD-aMCI and PD-CN were largely spatially overlapped with the memory-correlated FA values. Our findings demonstrated that the WM structural differences between PD-aMCI and PD-CN were mainly memory-related, and the influence of motor adjustment might indicate a common mechanism underlying both motor and memory impairment in PD-aMCI, possibly reflecting a predominant influence of dopaminergic neuropathology.

## 1 Introduction

Parkinson’s disease (PD) is often comorbid with mild cognitive impairment (MCI), a heterogeneous cognitive disorder characterized as mild deficits in various cognitive functions, with the prevalence varying between 25% and over 80% at different stages of PD [1]. According to the impaired cognitive functions, MCI can be classified into different subtypes with disparate neuroanatomical abnormalities [2, 3]. Similarly, PD-MCI can be classified into different cognitive impairment subtypes; among them, the amnestic subtype of PD-MCI (PD-aMCI) shows a higher progression rate to develop dementia [4, 5].

Several studies have investigated the neuroanatomical changes of PD-MCI [6–8]; however, few studies focused on any specific subtypes of PD-MCI [9]. To our knowledge, no study has examined the white matter (WM) changes of any specific PD-MCI subtype. Without specifying the subtypes of PD-MCI, several studies investigated the WM structural changes in PD-MCI, and the findings were inconsistent, possibly owing to the heterogeneous nature of PD-MCI [10–13]. Compared to cognitively normal PD patients, Agosta and colleagues found that PD-MCI showed a diffuse pattern of WM abnormalities [10], while two studies showed a localized WM decrease pattern for PD-MCI in the frontal, temporal and anterior cingulate WM bundles [11, 13]. There was also a report of no significant WM structural difference between PD-MCI and cognitively normal PD patients [12].

Our first aim would be to characterize the WM structural changes of PD-aMCI with cognitively normal PD patients (PD-CN) and normal controls on three diffusion tensor imaging (DTI) metrics, including fractional anisotropy (FA), axial diffusivity (AD) and radial diffusivity (RD). We employed AD and RD, instead of mean diffusivity (MD), as AD and RD could provide more detailed information on water diffusion directions than MD [14]. We used the voxel-based method for three DTI metrics, because it has the advantage of exploring the microstructural changes across the whole brain, including subcortical nuclei [15]. We also used the tract-based spatial statistics (TBSS) method to validate the voxel-based DTI analysis results, as the TBSS method could compensate some local registration errors of voxel-based analysis by restricting the analysis to the major WM tracts [16].

Normally, motor and memory are two separate brain functions with distinct brain structural substrates. However, in PD-aMCI there might be a common culprit for both motor and memory impairments. Evidence showed that the spread of dopaminergic neuropathology across diffuse brain regions coincided with the decline of motor and memory [17–19]. Yet, another line of evidence suggested that the accumulations of amyloid plaque and neurofibrillary tangle, the hallmarks of Alzheimer’s disease, had been specifically involved in the memory decline of PD patients, indicating a unique cholinergic pathological mechanism [8, 20, 21].

Driven by this knowledge, we considered that motor and memory impairments were both risk factors for the WM structural changes of PD-aMCI from PD-CN, and they may share a common pathological cause at certain extent, similar to the description of overlapping risk factors by Kraemer and colleagues [22]. As the adjustment of an overlapping risk factor could attenuate the relationship of another, we performed two sets of WM structural comparison analyses between PD-aMCI and PD-CN, without and then with a motor performance index controlled as a covariate. We hypothesized that some previously significant structural changes of PD-aMCI might be weakened to non-significant by the motor adjustment, which possible reflected the existence of a common dopaminergic mechanism underlying both motor and memory deficits in PD-aMCI. However, there might be some WM structures that could survive the motor adjustment, which might reflect the part of memory deficit that was uniquely affected by the cholinergic mechanism.

In summary, to characterize the WM structural changes of PD-aMCI and better understand its underlying neuropathological mechanisms, we performed two layers of analyses: 1) to compare the WM structural differences between PD-aMCI and PD-CN and normal controls using voxel-based and TBSS-based DTI metrics; 2) to exclude and then include the motor performance as a covariate in the WM structural comparison between PD-aMCI and PD-CN to discern the influences of two neuropathological mechanisms. Moreover, we performed the correlation analyses between memory and voxel-based WM measures in all PD patients, to localize the memory-associated WM structures.

## 2 Materials and methods

### 2.1 Participants

Thirty-six PD participants were randomly recruited from the Neurological Clinics of the First Affiliated Hospital of Fujian Medical University. The diagnosis of PD followed the UK Brain Bank criteria for idiopathic Parkinson’s disease [23], and the Hoehn and Yahr (H&Y) stages of PD were evaluated [24]. Motor function of the PD patients was assessed with the Movement Disorder Society (MDS) modified version of the Unified Parkinson’s Disease Rating Scale motor examination (UPDRS-III) [25]. We excluded the patients who were not at H&Y stage I-III or had been diagnosed with any of the following conditions: dementia based on DSM-IV criteria [26], a history of brain surgery, stroke, epilepsy, multiple sclerosis, progressive malignancy (active cancer or receiving radiotherapy for cancer, other than prostate or mild skin cancer), schizophrenia, bipolar disorder, and developmental disability. We also excluded the PD patients who had a Mini-Mental State Examination score (MMSE) ≤ 24 [27].

We recruited 20 healthy older adults, who did not have any known or suspected history of neurological illness or psychiatric impairments including MCI. The normal controls (n = 20) and PD patients (n = 36) were matched on age, gender, years of education and MMSE.

This study was approved by the ethics committee of Fujian Medical University and written informed consents were obtained from all participants.

### 2.2 MCI classification

The Chinese version of Repeatable Battery for the Assessment of Neuropsychological Status (RBANS) [28] were administered by trained medical graduates to assess cognitive functions of the participants. The RBANS consists of twelve subtests that evaluate five cognitive domains, which are immediate memory (list learning and story memory), delayed memory (list recall, list recognition, story recall, and figure recall), language (picture naming and semantic fluency), attention (digit span and coding), and visuospatial function (figure copy and line orientation) [29]. The RBANS index score of each cognitive domain was the average T-score of the subtests that consist of that domain; raw score of each subtest of each individual was firstly standardized to z-score with the mean and standard deviation of that subtest in all participants, and then transformed to T-score with a mean of 100 and standard deviation of 15.

In line with the recommendations of the Movement Disorders Society on PD-MCI [30], the diagnosis of aMCI in PD patients was operationalized by 1) no significant impairment in activities of daily living according to medical history; 2) a report of memory complaints, either from the participants or their informant; and 3) memory impairment defined by the performance on at least two tests in the immediate and/or delayed memory domains≤ -1.5 standard deviation (SD) of the published normative values [31].

Seventeen PD patients met the criteria for PD-aMCI. Nineteen PD patients did not fulfill the criteria for MCI and were classified as cognitively normal PD (PD-CN). No significant differences were found between PD-aMCI and PD-CN on age, gender, years of education, disease duration, H&Y stage, UPDRS-III, Levodopa equivalent daily dose and MMSE.

### 2.3 Image acquisition

MRI scans of all participants were acquired on a 3.0 T SIEMENS Magnetom Verio MRI scanner (Siemens Medical Solutions, Erlangen, Germany) at the Department of Radiology of the First Affiliated Hospital of Fujian Medical University. All PD participants and healthy controls underwent a standardized brain MRI protocol. The PD participants were assessed “ON” their usual dopaminergic medication. DTI scans were acquired using a single-shot echo-planar imaging-based sequence with the following parameters: TR = 4316 ms, TE = 95 ms, flip angle = 90°, voxel size = 2 x 2 x 4.55 mm^3^, acquisition matrix = 128 x 128, FOV = 256 x 256 mm^2^, 62 non-linear diffusion weighting directions with b = 1,000 s/mm^2^ and one image without diffusion weighting (*i*.*e*., b = 0 s/mm^2^).

### 2.4 Image processing

After visually inspecting MRI scans for structural abnormalities, the DTI dataset was processed with the FSL 5.0 package (http://www.fmrib.ox.ac.uk/fsl/). The raw DTI images were firstly undergone head motion, eddy current correction, and skull stripping; then the diffusion tensor was reconstructed by fitting a diffusion tensor model for each image [16]. Diffusion metrics of FA, AD, and RD were calculated from the diffusion-weighted images. Subsequently, the individual voxel-wise maps of FA, AD, and RD were spatially transformed to the MNI standard space in a 2 x 2 x 2 mm spatial resolution; and then finally smoothed with a 6-mm FWHM Gaussian kernel for voxel-based DTI statistical analysis. The FA images were then averaged to generate a mean FA image, which was used to create an FA skeleton by selecting the voxels with the locally maximal FA values. The generated FA skeleton was then thresholded at FA > 0.2 to minimize the partial volume effect and cross-subject misregistration [15]. Subsequently, the diffusion metrics of FA, AD, and RD were individually projected onto that skeleton, and the resultant maps of FA, AD, and RD on the skeleton were fed into the TBSS-based DTI statistical analyses.

### 2.5 Statistical analysis

We compared the differences between PD-aMCI and PD-CN, or between PD-CN and normal controls, on voxel-based DTI metrics (FA, AD, and RD). The analysis between PD-aMCI and PD-CN could reveal the WM structural changes associated with aMCI in PD patients, while the analysis between PD-CN and normal controls could exhibit the WM structural changes associated with PD. The controlled covariates included age, gender, and disease duration (only for the comparison between PD-aMCI and PD-CN). We also performed TBSS-based group comparison analyses, controlled for the same covariates to validate the results of voxel-based analysis. UPDRS-III was then added in the comparison analyses between PD-aMCI and PD-CN on voxel-based DTI measures as a covariate.

Furthermore, correlation analyses were performed between two memory scores (immediate memory and delayed memory) and voxel-based DTI measures in all PD participants. The controlled covariates were age, gender, and disease duration. We then used the conjunction overlay method [32] to overlay the structural correlation map of memory upon the structural difference map between PD-aMCI and PD-CN, and the common areas from the two maps would indicate the correspondence. UPDRS-III was then added as a covariate in the WM structural correlation analyses of memory, to verify if the motor adjustment could also affect the memory-WM relationship.

In addition, we performed the correlation analyses for language, attention, visuospatial function, and UPDRS-III with voxel-based DTI measures in all PD participants, controlled for age, gender, and disease duration. The partial correlations between UPDRS-III and each cognitive performance were also examined in all PD patients, controlled for age, gender, and disease duration.

All statistical analyses with WM structural measures were implemented in FSL 5.0, and the correction for multiple comparisons employed the threshold-free cluster-enhancement (TFCE) method, which is a non-parametric permutation test [33, 34]. We performed 5000 permutations and set the significance threshold at p<0.05 (FWE-corrected). The supra-threshold clusters of DTI metrics were superimposed on a series of brain slices in the MNI 152 T1 brain template. The significant results on TBSS-based diffusion metrics were dilated to enhance visualization.

## 3 Results

The demographic characteristics and cognitive performance for different PD groups and normal controls were shown in Table 1.

**Table 1:** Demographic characteristics and cognitive performance for different PD groups and normal controls.

Mean ± SD or %	Normal controls (n = 20)	PD-CN (n = 19)	p-value^a^	PD-aMCI (n = 17)	p-value^b^
Age, years	59.5±6.2	61.3±6.9	0.41	64.9±5.9	0.11
Gender (% male)	80%	78.9%	0.94	88.2%	0.46
Education, years	9.3±2.2	10.3±3.3	0.29	9.6±3.8	0.60
Disease duration, years	/	5.9±3.4	/	7.6±4.9	0.23
Hoehn and Yahr stage	/	1.5±0.8	/	1.9±0.8	0.14
UPDRS-III score	/	17.7±9.7	/	24.4±11.2	0.07
Levodopa equivalent daily dose (mg)	/	917.0±144.6	/	942.1±120.6	0.58
MMSE score	29.5±0.4	29.4±0.8	0.63	28.5±1.4	0.09
**RBANS index scores**					
Immediate memory	113.6±7.9	108.8±10.2	0.12	74.2±8.1	<0.001
Delayed memory	112.1±9.7	109.3±9.0	0.38	75.4±10.1	<0.001
Attention	106.2±10.5	100.5±7.6	0.07	92.2±11.2	0.02
Language	104.8±7.1	99.8±11.3	0.12	94.6±7.3	0.11
Visuospatial function	102.3±11.3	100.6±13.2	0.68	96.6±12.6	0.36

PD = Parkinson’s disease; PD-aMCI = amnestic mild cognitive impairment in PD; PD-CN = cognitively normal PD patient; UPDRS-III = the Movement Disorder Society modified version of the Unified Parkinson’s Disease Rating Scale–part III for motor examination; MMSE = Mini-Mental State Examination. The differences between PD-CN and normal controls, as well as between PD-aMCI and PD-CN in the variables were examined, and the significance values were indicated by p-value^a^ and p-value^b^, respectively.

**Fig 1:**
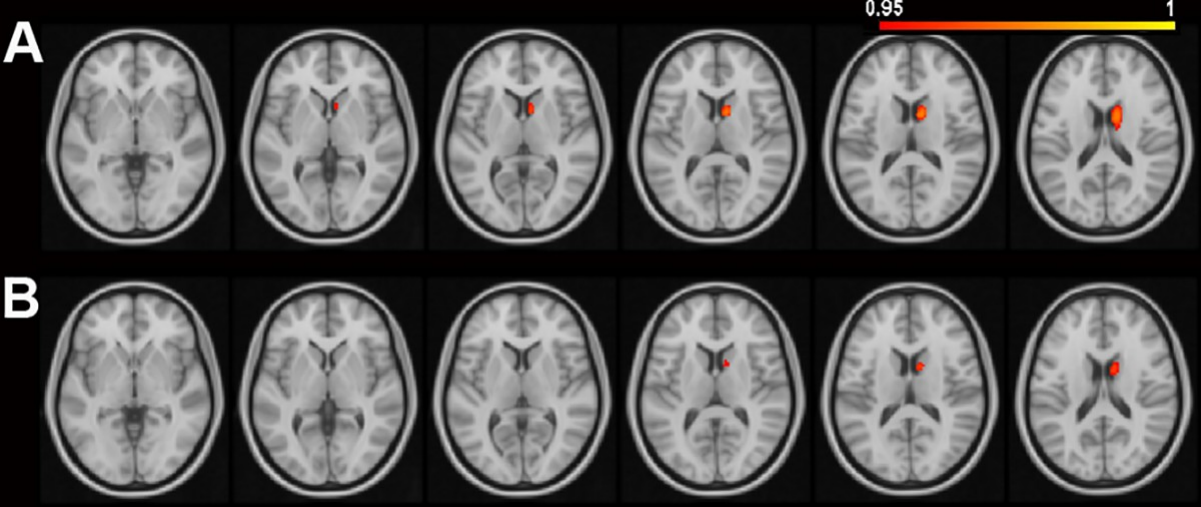
DTI measure differences between PD-CN and normal controls. Compared to normal controls, PD-CN showed significantly higher voxel-based DTI values in the caudate. The color bar indicates the 1-p value ranging from 0.95 to 1. Higher axial diffusivity values A) and radial diffusivity values B) in PD-CN were shown on the axial slices ranging from -2 mm to 18 mm at z-axis, with an interval of 4 mm (from bottom to top).

**Fig 2:**
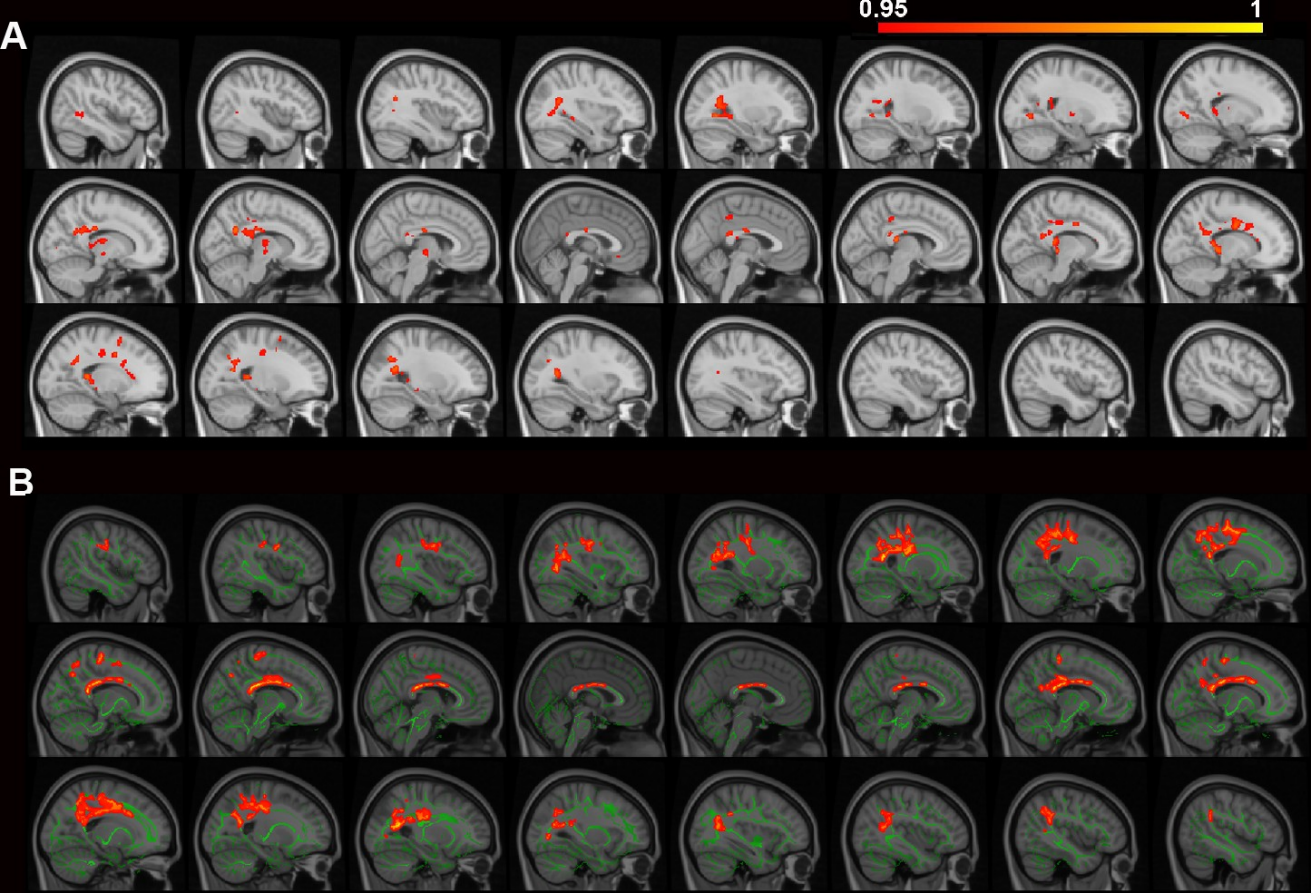
Differences in voxel-based and TBSS-based FA values between PD-aMCI and PD-CN. Compared to PD-CN, PD-aMCI showed smaller fractional anisotropy (FA) values. The smaller voxel-based FA values A) and TBSS-based FA values B) were located in the brain areas superimposed on a series of sagittal slices ranging from 46 mm to -46 mm at x-axis (from right to left), with an interval of 4 mm. The color bar indicates the 1-p value ranging from 0.95 to 1. In B), the significant TBSS-based FAs (in red) were dilated to enhance visualization, overlaying on the white matter skeleton (in green).

Compared to normal controls, PD-CN patients showed higher AD and RD values in the right caudate (Table 2, Fig 1A and 1B). Compared to PD-CN, PD-aMCI patients showed lower FA values in the corpus callosum (splenium and body), posterior thalamic radiation, posterior corona radiata, tapetum, cingulum (cingulate gyrus) in the bilateral hemispheres, the left superior corona radiata and fornix (crux), and the right superior longitudinal fasciculus (Table 2 and Fig 2A). TBSS-based FA values were compared between PD-aMCI and PD-CN: PD-aMCI showed lower FA values in the WM tracts of the corpus callosum (body and splenium), superior and inferior longitudinal fasciculus, cingulum (cingulate gyrus), and inferior fronto-occipital fasciculusin the bilateral hemispheres (Table 2 and Fig 2B). By visualizing Fig 2A and 2B, a similar spatial distribution was noted in the two FA-difference maps of PD-aMCI. No significant GM differences were found between PD-aMCI and PD-CN, as well as between PD-CN and controls.

**Table 2:** Differences in DTI metrics between PD-aMCI and PD-CN and between PD-CN and normal controls.

Comparison	Metrics	Cluster	Peak voxels				
		Size	MNI coordinates			1-p value	Anatomical location
			X	Y	Z		
PD-CN	Voxel/AD	317	12	4	16	0.98	R caudate
> NC	Voxel/RD	83	12	4	16	0.96	R caudate
PD-aMCI	Voxel/FA	810	10	-39	20	0.96	R corpus callosum splenium
< PD-CN			-3	-17	24	0.96	R corpus callosum body
			-8	-51	16	0.96	L cingulum (cingulate gyrus)
			-28	-56	17	0.97	L posterior thalamic radiation
			-19	-50	33	0.96	L posterior corona radiata
			-29	-54	13	0.97	L tapetum
			10	-45	23	0.95	R cingulum (cingulate gyrus)
		744	33	-52	18	0.97	R posterior thalamic radiation
			32	-49	8	0.95	R tapetum
			32	-53	21	0.97	R posterior corona radiata
			36	-53	18	0.96	R superior longitudinal fasciculus
		318	-18	-1	38	0.96	L superior corona radiata
		313	-25	-25	-10	0.95	L fornix
	TBSS/FA	9124	-12	-9	30	0.97	L cingulum body
			11	-25	28	0.98	R cingulum body
			-37	-54	15	0.97	L superior longitudinal fasciculus
			35	-12	34	0.96	R superior longitudinal fasciculus
			-9	-29	35	0.97	L cingulum (cingulate gyrus)
			9	-6	33	0.97	R cingulum (cingulate gyrus)
			-27	-58	19	0.97	L inferior longitudinal fasciculus
			28	-50	19	0.97	R inferior longitudinal fasciculus
			32	-62	1	0.96	R inferior fronto-occipital fasciculus
		41	-28	-74	3	0.95	L inferior fronto-occipital fasciculus

PD = Parkinson’s disease; PD-aMCI = amnestic mild cognitive impairment in PD; PD-CN = cognitively normal PD patient; AD = axial diffusivity; RD = radial diffusivity; FA = fractional anisotropy. TBSS = Tract-based spatial statistics.

Voxel-based DTI metrics (FA, AD, and RD) were compared between PD-CN and normal controls, as well as between PD-aMCI and PD-CN. TBSS-based FA values were also compared between PD-aMCI and PD-CN. The significance level was set at p<0.05 (FWE-corrected). The controlled covariates included age, gender, and disease duration (only for the comparison between PD-aMCI and PD-CN).

**Fig 3:**
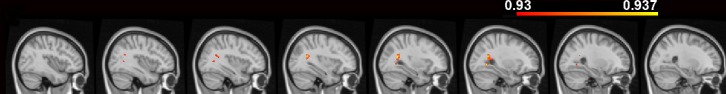
Adjusting for UPDRS-III in the comparison of voxel-based FA between PD-aMCI and PD-CN. UPDRS-III was adjusted in the comparison analysis of voxel-based FA between PD-aMCI and PD-CN, along with age, gender, and disease duration. At the threshold of p<0.07 (FWE-corrected), smaller FA values in PD-aMCI were shown on the sagittal slices ranging from 40 mm to 26 mm at x-axis (from right to left), with an interval of 2 mm.

The adjustment of UPDRS-III in the comparison between PD-aMCI and PD-CN weakened all FA differences to non-significant at the threshold of p<0.05 (FWE-corrected); however, some FA differences could survive at the threshold of p<0.07 (FWE-corrected), located in the right posterior thalamic radiation, posterior corona radiata, and tapetum (Table 3 and Fig 3).

**Table 3:** Controlling for UPDRS-III in the comparison of FA between PD-aMCI and PD-CN.

Analysis	Cluster size	Peak voxels				
		MNI coordinates			1-p value	Anatomical location
		X	Y	Z		
PD-aMCI	74	33	-51	17	0.94	R posterior thalamic radiation
< PD-CN		30	-52	19	0.94	R posterior corona radiata
		31	-50	16	0.93	R tapetum

PD = Parkinson’s disease; PD-aMCI = amnestic mild cognitive impairment in PD; PD-CN = cognitively normal PD patients; UPDRS-III = the Unified Parkinson’s Disease Rating Scale–part III for motor examination; FA = fractional anisotropy.

Voxel-based FA values were compared between PD-aMCI and PD-CN, controlled for age, gender, disease duration, and UPDRS-III. The significance level was set at p<0.07 (FWE-corrected).

**Fig 4:**
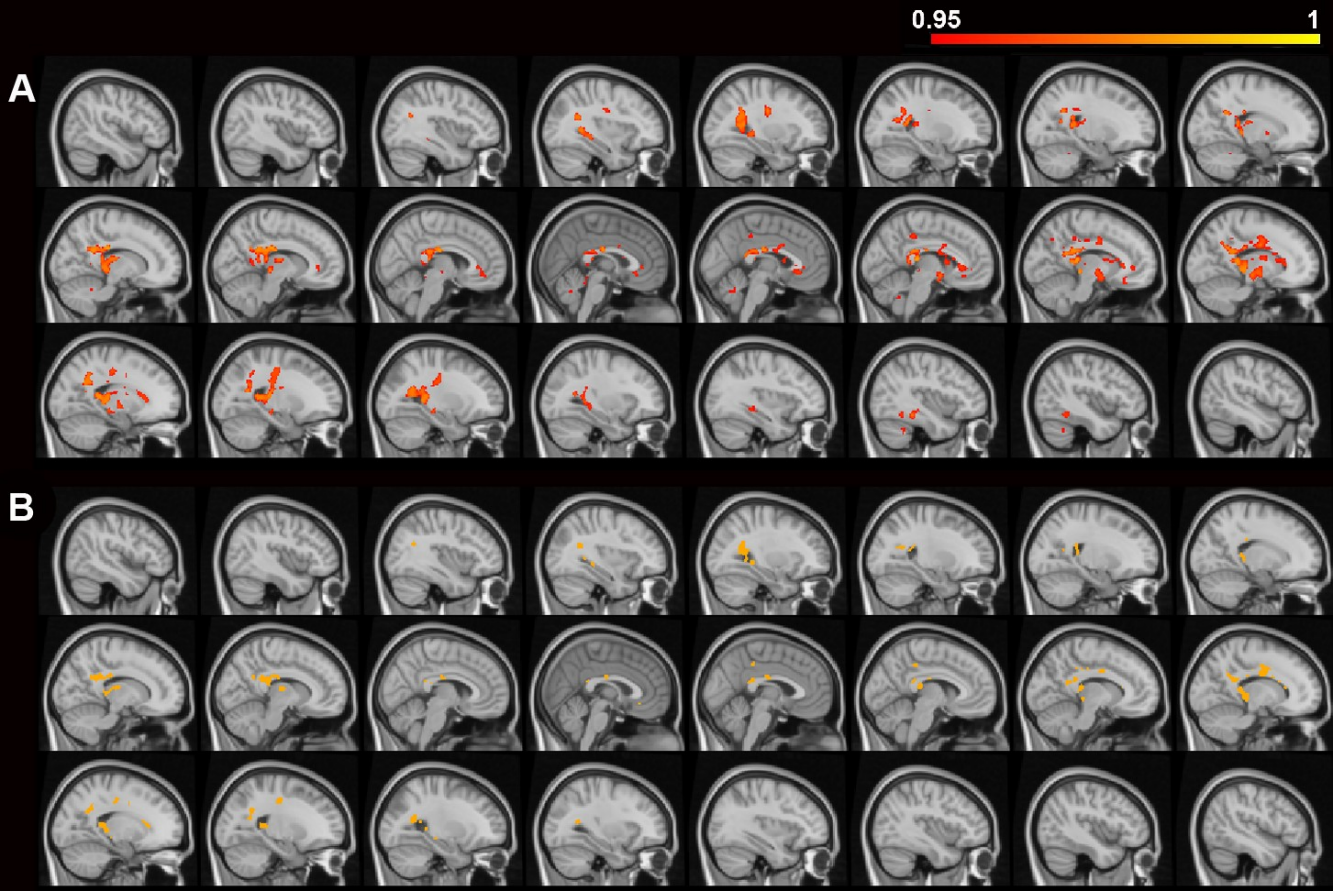
Voxel-based FA correlates of memory in PD patients and the conjunction overlay. Voxel-based fractional anisotropy (FA) values that were significantly correlated with delayed memory in all PD patients were superimposed on a series of sagittal slices in the brain template (from right to left). The color bar indicates the 1-p value ranging from 0.95 to 1. A) The positive FA correlates of delayed memory were shown on the slices ranging from 46 mm to -46 mm at x-axis, with an interval of 4 mm. The conjunction overlay analysis showed B) the positive FA correlates of delayed memory were overlapped with the FA differences between PD-aMCI and PD-CN, and the common brain areas (in orange) were superimposed on the slices ranging from 46 mm to -46 mm at x-axis, with an interval of 4 mm.

The structural correlation analyses of memory in all PD patients showed that delayed memory was positively correlated with voxel-based FA values in diffuse areas (Table 4, Fig 4A). The conjunction overlay demonstrated that the FA correlates of delayed memory were mostly overlapped with the FA differences between PD-aMCI and PD-CN, including the bilateral corpus callosum (splenium and body), cingulum (cingulate gyrus), posterior corona radiata, tapetum, the left fornix and superior corona radiata, as well as the right posterior thalamic radiation and superior longitudinal fasciculus (Fig 4B). The adjustment of UPDRS-III weakened all previously significant FA correlates of delayed memory to non-significant at the threshold of p<0.05 (FWE-corrected); however, a few FA correlates could survive at the threshold of p< 0.07 (FWE-corrected), located in the bilateral corpus callosum (body and splenium), right posterior cingulum, posterior thalamic radiation, posterior corona radiata, and tapetum (S1 Table and S1 Fig).

**Table 4:** Correlations between voxel-based FA and memory in PD patients.

Cognition	Cluster size	Peak voxels				
		MNI coordinates			1-p value	Anatomical location
		X	Y	Z		
Delayed	3792	5	-18	26	0.98	R corpus callosum body
memory		-13	-42	23	0.98	L corpus callosum splenium
		29	-47	22	0.97	R posterior corona radiata
		-26	-54	21	0.97	L posterior corona radiata
		-22	-21	34	0.96	L superior corona radiata
		10	-41	28	0.98	R cingulum (cingulate gyrus)
		-10	-22	36	0.95	L cingulum (cingulate gyrus)
		27	-34	-1	0.96	R fornix (crux)
		-24	-24	-9	0.96	L fornix (crux)
		29	-46	16	0.97	R tapetum
		-28	-53	17	0.97	L tapetum
		-22	-24	16	0.97	L internal capsule (posterior limb)
	79	30	-13	30	0.97	R superior corona radiata

FA = fractional anisotropy; PD = Parkinson’s disease

Voxel-based FA values were correlated with the performance of different cognitive domains in 36 PD patients. The controlled covariates included age, gender, and disease duration. The significance level was set at p<0.05 (FWE-corrected). Only delayed memory and attention were significantly positively correlated with FA.

Attention was positively correlated with the FA values in the right corpus callosum splenium and posterior corona radiata (S2 Table). The FA correlates of attention shared a small extent of common area (only 4 voxels) with the FA differences between PD-aMCI and PD-CN (not shown). No significant structural correlations were found for other cognitive performance and UPDRS-III in all PD patients. The correlations of UPDRS-III with different cognitive functions were: attention (r = -0.35, p = 0.047), delayed memory (r = -0.27, p = 0.12), immediate memory (r = -0.25. p = 0.15), language (r = -0.15, p = 0.40), and visuospatial function (r = -0.29, p = 0.10).

## 4 Discussion

### 4.1 FA changes in PD-aMCI

No prior study examined the WM changes of PD-aMCI. Using voxel-based and TBSS-bases analyses, we demonstrated a diffuse pattern of FA decreases of PD-aMCI compared to PD-CN, located in the corpus callosum (splenium and body), posterior thalamic radiation, posterior corona radiata, tapetum, and cingulum (cingulate gyrus). A few studies investigated the WM changes of PD-MCI, without specifying the subtype of MCI, and revealed significant FA decreases across different WM tracts in PD-MCI, compared to PD-CN [10, 13]. Given that a lower FA value indicates decreased fiber integrity induced by demyelination [14, 35, 36], our findings possibly suggested that PD-aMCI might have an extensive fiber integrity disruption, relative to PD-CN.

### 4.2 FA changes of PD-aMCI mostly overlapped with the FA correlates of delayed memory

By the conjunction overlay, we demonstrated that the FA difference between PD-aMCI and PD-CN were mostly overlapped with the FA correlates of delayed memory. This finding indicated that decreased fiber integrity in PD-aMCI was mainly contributed by the WM abnormality associated with memory decline. In healthy older adults, the WM measures associated with memory were mainly localized in the fornix and cingulum [37–39]. However, in PD patients, several studies showed a diffuse WM correlation pattern with memory, which have been demonstrated in the fornix, cingulum, corpus callosum and posterior corona radiate, consistent with our findings [40–42].

### 4.3 AD and RD changes in PD-CN

PD-CN showed significantly higher AD and RD values than normal controls in the right caudate, whilst no difference in FA values. AD and RD estimate the diffusivity of water molecule along and perpendicular to the direction of WM tracts, respectively [14]. Thus, when water diffusivity in different directions is proportional, a minimal change in FA would be expected [43]. Such patterns of changes in AD, RD, and FA have been suggested as an indication of enlarged extracellular space, which could be due to neurodegeneration involving neuronal death and fiber shrinkage [44–46].

The degeneration of dopaminergic neurons in the basal ganglia is a pathological feature of PD [17, 18]. Caudate is an essential component of the cortico-basal ganglia-thalamo-cortical pathways to regulate movement [47]. The changes of MD and FA in the caudate have been reported in PD patients [48, 49]. Moreover, several studies demonstrated the dopaminergic denervation, GM atrophy, and morphological deformation in the caudate in PD patients [50–53]. These lines of evidence were consistent with our findings on the increases of AD and RD in the caudate, suggesting a possible neurodegeneration in this area in PD patients.

### 4.4 Possible neuropathological mechanisms of PD-aMCI

Our study showed that the adjustment of UPDRS-III could weaken the originally significant FA differences between PD-aMCI and PD-CN and FA correlates of memory to non-significant. Motor and memory are two distinct brain functions with diverse neural substrates, which are normally not related. However, in PD-aMCI, the impairments in both functions might be subject to a common dopaminergic pathological influence [17–19]. Meanwhile, evidence suggested that the cholinergic mechanism was also involved in the PD-aMCI [8, 20, 21].

The extensive influence of motor adjustment as shown in our results might reflect a predominant influence of dopaminergic neuropathology [19]. However, we also noted a tendency towards significance for some voxel-based FA differences between PD-aMCI and PD-CN, which were located in the posterior cingulum, posterior thalamic radiation, and tapetum. These regions were adjacent to the fornix and hippocampal formation, which might suggest the specific influence of cholinergic neuropathology on memory [37–39].

Our findings were in accordance with the theory of dual neuropathological mechanisms in PD-aMCI. It has been suggested that the two neuropathological mechanisms could drive synergistically neurodegenerative processes to undermine brain structural integrity of PD-aMCI and accelerate its conversion to dementia [8, 54].

### 4.5 WM structural correlation analyses of attention, language, visuospatial function in all PD patients

Only attention showed positive correlations with the FAs in the right corpus callosum splenium and posterior corona radiata, while language and visuospatial function showed null correlations. These results were expected, given that PD-aMCI and PD-CN had no significant differences on language and visuospatial function, which lead to a small inter-individual variation in all PD participants and reduced likelihood for a significant cognitive correlation. Moreover, different from memory, the FA correlates of attention had a small spatial overlap (4 voxels) with the FA differences between PD-aMCI and PD-CN, suggesting a limited contribution of attention-related WM changes to the WM abnormality of PD-aMCI.

### 4.6 Limitation

Despite that the number of our PD-aMCI patients is reasonable comparing to prior neuroimaging studies on PD-MCI, our sample size is small. To alleviate the concern on the robustness of the results, we performed the TBSS-based FA analysis as a complementary method to validate the results of voxel-based FA analysis. The results indicated a similar distribution pattern between the voxel-based and TBSS-based FA difference maps. Moreover, we employed the threshold-free cluster-enhancement (TFCE) method, which has been recommended as a stringent statistical method in voxel-wise analysis [33, 34].

### 4.7 Conclusion

No previous neuroimaging studies have examined the WM structural changes of a specific PD-MCI subtype, PD-aMCI. Our study showed a diffuse FA decrease pattern for PD-aMCI compared to PD-CN. However, most FA difference between PD-aMCI and PD-CN could be weakened by the adjustment of motor performance, which might indicate a predominant influence of dopaminergic neuropathology. Yet, some FA differences of PD-aMCI adjacent to the fornix and hippocampal formation showed a tendency to survive the motor adjustment, which might reflect a memory-specific influence by the cholinergic neuropathological mechanism.

## Supporting information

S1 Table Controlling for UPDRS-III in the correlation analysis between delayed memory and voxel-based FA in all PD patients. (DOCX)

S2 Table Correlations between voxel-based FA and attention in PD patients. (DOCX)

S1 Fig Adjusting for UPDRS-III in the correlation analysis between delayed memory and voxel-based FA in all PD patients. UPDRS-III was controlled in the correlation analysis between voxel-based FA and delayed memory in all PD patients, along with age, gender, and disease duration. At the threshold of p<0.07 (FWE-corrected), the FA correlates of delayed memory were superimposed on the sagittal slices ranging from 34 mm to -26 mm at x-axis (from right to left), with an interval of 4 mm. The color bar indicates the 1-p value ranging from 0.93 to 0.95.
(TIF)
