# Supplementary material for: Amnestic mild cognitive impairment in Parkinson’s disease: White matter structural changes and mechanisms
Source: PLoS One. 2019 Dec 12;14(12):e0226175. doi: 10.1371/journal.pone.0226175 (PMC6907797; doi:10.1371/journal.pone.0226175)
Supplement: S1 Table — (DOCX) [file pone.0226175.s001.docx]

**S1 Table.** **Controlling for UPDRS-III in the correlation analysis between delayed memory and voxel-based FA in all PD patients**

| Cognition | Cluster size | Peak voxels | | | | |
| --- | --- | --- | --- | --- | --- | --- |
|  |  | MNI coordinates | | | 1-p value | Anatomical location |
|  |  | X | Y | Z |  |  |
| Delayed | 156 | 4 | -18 | 25 | 0.94 | R corpus callosum body |
| memory |  | 14 | -45 | 25 | 0.94 | R corpus callosum splenium |
|  |  | 10 | -44 | 25 | 0.93 | R cingulum |
|  | 63 | -10 | -16 | 25 | 0.94 | L corpus callosum body |
|  |  | -8 | -34 | 24 | 0.93 | L corpus callosum splenium |
|  | 50 | 32 | -48 | 22 | 0.93 | R posterior corona radiata |
|  |  | 30 | -53 | 18 | 0.93 | R posterior thalamic radiation |
|  | 51 | 32 | -47 | 8 | 0.94 | R tapetum |

PD = Parkinson’s disease; UPDRS-III = the Unified Parkinson’s Disease Rating Scale – part III for motor examination.

UPDRS-III was controlled in the correlation analysis between voxel-based FA and delayed memory in all PD patients, along with age, gender, and disease duration. The FA correlates of delayed memory at the threshold of p<0.07 (FWE-corrected) were shown.
