# Supplementary material for: Amnestic mild cognitive impairment in Parkinson’s disease: White matter structural changes and mechanisms
Source: PLoS One. 2019 Dec 12;14(12):e0226175. doi: 10.1371/journal.pone.0226175 (PMC6907797; doi:10.1371/journal.pone.0226175)
Supplement: S2 Table — (DOCX) [file pone.0226175.s002.docx]

**S2 Table. Correlations between voxel-based FA and attention in PD patients**

| Cognition | Cluster size | Peak voxels | | | | |
| --- | --- | --- | --- | --- | --- | --- |
|  |  | MNI coordinates | | | 1-p value | Anatomical location |
|  |  | X | Y | Z |  |  |
| Attention | 287 | 18 | -48 | 25 | 0.97 | R corpus callosum splenium |
|  |  | 22 | -49 | 41 | 0.97 | R posterior corona radiata |

FA = fractional anisotropy; PD = Parkinson’s disease

Voxel-based FA values were correlated with the performance of different cognitive domains in 36 PD patients. The controlled covariates included age, gender, and disease duration. The significance level was set at p<0.05 (FWE-corrected). Only attention was significantly positively correlated with FA.
