# Supplementary figures and images for: Amnestic mild cognitive impairment in Parkinson’s disease: White matter structural changes and mechanisms
Source: PLoS One. 2019 Dec 12;14(12):e0226175. doi: 10.1371/journal.pone.0226175 (PMC6907797; doi:10.1371/journal.pone.0226175)

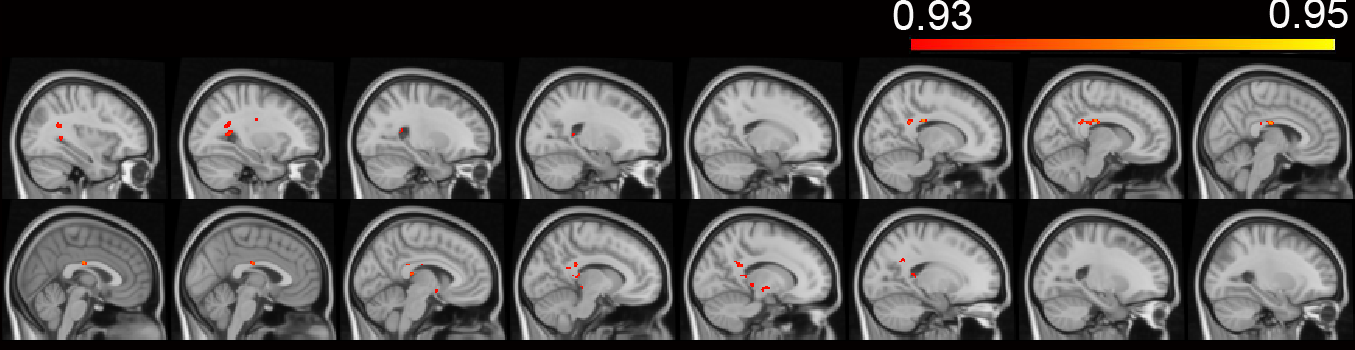

Supplement: S1 Fig — UPDRS-III was controlled in the correlation analysis between voxel-based FA and delayed memory in all PD patients, along with age, gender, and disease duration. At the threshold of p<0.07 (FWE-corrected), the FA correlates of delayed memory were superimposed on the sagittal slices ranging from 34 mm to -26 mm at x-axis (from right to left), with an interval of 4 mm. The color bar indicates the 1-p value ranging from 0.93 to 0.95. (TIF) [file pone.0226175.s003.tif]
